# The Phases of Living Evidence Synthesis Using AI: Living Evidence Synthesis (Version 1)

**DOI:** 10.2196/76130

**Published:** 2026-01-27

**Authors:** Xuping Song, Zhenjie Lian, Rui Wang, Ruixin Li, Zhenzhen Yang, Xufei Luo, Lei Feng, Zhiming Ma, Zhen Pu, Qi Wang, Long Ge, Caihong Li, Yaolong Chen, Kehu Yang, John Lavis

**Affiliations:** 1 School of Public Health, Lanzhou University, No. 222 South Tianshui Road, Lanzhou, Lanzhou, Gansu, 753000, China, +86 13893117077, +86 13893117077; 2 The Centre of Evidence-based Social Science, Lanzhou University, Lanzhou, Gansu, China; 3 Key Laboratory of Evidence Based Medicine & Knowledge Translation of Gansu Province, Lanzhou, Gansu, China; 4 WHO Collaborating Centre for Guideline Implementation and Knowledge Translation, Lanzhou, Gansu, China; 5 Dingxi Center for Disease Control and Prevention, Dingxi, Gansu, China; 6 Evidence-Based Medicine Center, School of Basic Medicine, Lanzhou University, Lanzhou, Gansu, China; 7 School of Public Health, University of Hong Kong, Hong Kong, China; 8 School of Information Science and Engineering, Lanzhou University, Lanzhou, Gansu, China; 9 Department of Health Research Methods, Evidence, and Impact, McMaster Health Forum, McMaster University, Hamilton, ON, Canada

**Keywords:** accuracy, artificial intelligence, efficiency, living evidence synthesis, phases, semiautomated tools, utility

## Abstract

**Background:**

Living evidence (LE) synthesis refers to the method of continuously updating systematic evidence reviews to incorporate new evidence. It has emerged to address the limitations of the traditional systematic review process, particularly the absence of or delays in publication updates. The emergence of COVID-19 accelerated the progress in the field of LE synthesis, and currently, the applications of artificial intelligence (AI) in LE synthesis are expanding rapidly. However, in which phases of LE synthesis should AI be used remains an unanswered question.

**Objective:**

This study aims to (1) document the phases of LE synthesis where AI is used and (2) investigate whether AI improves the efficiency, accuracy, or utility of LE synthesis.

**Methods:**

We searched Web of Science, PubMed, the Cochrane Library, Epistemonikos, the Campbell Library, IEEE Xplore, medRxiv, COVID-19 Evidence Network to support Decision-making, and McMaster Health Forum. We used Covidence to facilitate the monthly screening and extraction processes to maintain the LE synthesis process. Studies that used or developed AI or semiautomated tools in the phases of LE synthesis were included.

**Results:**

A total of 24 studies were included, including 17 on LE syntheses, with 4 involving tool development, and 7 on living meta-analyses, with 3 involving tool development. First, a total of 34 AI or semiautomated tools were involved, comprising 12 AI tools and 22 semiautomated tools. The most frequently used AI or semiautomated tools were machine learning classifiers (n=5) and the Living Interactive Evidence synthesis platform (n=3). Second, 20 AI or semiautomated tools were used for the data extraction or collection and risk of bias assessment phase, and only 1 AI tool was used for the publication update phase. Third, 3 studies demonstrated the improvement in efficiency achieved based on time, workload, and conflict rate metrics. Nine studies applied AI or semiautomated tools in LE synthesis, obtaining a mean recall rate of 96.24%, and 6 studies achieved a mean *F*_1_-score of 92.17%. Additionally, 8 studies reported precision values ranging from 0.2% to 100%.

**Conclusions:**

AI and semiautomated tools primarily facilitate data extraction or collection and risk of bias assessment. The use of AI or semiautomated tools in LE synthesis improves efficiency, leading to high accuracy, recall, and *F*_1_-scores, while precision varies across tools.

## Introduction

Evidence synthesis refers to an approach where data across studies are identified and combined to gain a clearer understanding of a body of research [1]. There is typically a significant gap between the time when a search is performed and the time when the results are published, often exceeding a year [2]. Furthermore, only a limited number of reviews are updated once they have been published [3]. This process can result in missing evidence, potentially affecting the accuracy of the findings. The approach of living evidence (LE) synthesis has been developed to address this challenge.

The method of constantly updating a systematic synthesis of evidence to incorporate newly available evidence is known as LE [4]. Elliott et al [5] developed the basis of the LE model in 2014, which effectively incorporates and summarizes new evidence. The LE synthesis process includes 4 phases: database searching and eligibility assessment, data extraction or collection and risk of bias assessment, synthesis and analysis, and publication update [6]. It has also been adapted in areas such as network meta-analysis and guidelines. The onset of COVID-19 increased the incentive to use LE [7]. Unlike traditional evidence synthesis, which requires the redeployment of significant resources for updates, the maintenance of an LE synthesis can require more modest resources [8]. However, LE synthesis that focuses on evolving topics may have a reduced reliability compared to traditional evidence synthesis. The incorporation of artificial intelligence (AI) techniques has the potential to enhance the reliability of LE synthesis by, for example, leveraging advanced algorithms to continuously assess and filter the most relevant and high-quality evidence [9].

The field of AI, which encompasses machine learning, deep learning, natural language processing, data mining, image recognition, and computer vision, to name a few, has the potential to enhance the efficiency of LE synthesis [10,11]. In 2013, Adams et al [11] indicated that leveraging AI to automate the LE synthesis procedures could simplify the regular updating and maintenance of evidence. The development of AI systems, particularly AI based on large language models (LLMs), such as the generative pretrained transformer, has significantly advanced natural generative language systems [12]. Various AI-driven tools have been developed for different phases of LE synthesis, such as crowdsourcing and task-sharing platforms like HDAS [13]. However, the performance of the AI techniques and the phases of LE synthesis where AI is used remain unclear.

Overall, the objectives of this review are (1) to conduct a review analyzing the phases of LE synthesis that use AI and (2) to explore whether AI can improve the efficiency, accuracy, or utility of LE synthesis.

## Methods

This is the first version of an LE synthesis. The Preferred Reporting Items for Systematic Reviews and Meta-Analyses 2020 statement for living systematic reviews (PRISMA-LSR; Checklist 1) was used as a guide for reporting this LE synthesis [14]. The review has been registered in the Open Science Forum [15].

### Search Strategy

We systematically searched the Web of Science, PubMed, the Cochrane Library, Epistemonikos, the Campbell Library, IEEE Xplore, medRxiv, COVID-19 Evidence Network to support Decision-making, and McMaster Health Forum for publications up to April 2, 2025. The details of the search strategy used can be found in Table S1 in Multimedia Appendix 1. We subscribed to the Web of Science, PubMed, the Cochrane Library, the Campbell Library, and IEEE Xplore for monthly dynamic updates and used Covidence to facilitate the screening and extraction processes for maintaining an LE synthesis. We plan to conduct living updates for a 12-month period (from April 2025 to April 2026). The final update is scheduled for April 2, 2026, after which we will assess whether to retire the living mode based on the following established triggers: (1) evidence on “the AI application in LE synthesis” has reached conclusiveness, (2) the topic no longer holds decision-making value for the field, (3) no new eligible studies emerge during the 12-month update period, or (4) subsequent resource or funding support is unavailable [16,17].

### Inclusion and Exclusion Criteria

First, the LE synthesis includes living systematic review, living meta-analysis, living network meta-analysis, living guideline, living scoping review, living overview, living umbrella review, and living mapping. In this review, the types of included studies were classified into 2 categories based primarily on whether a meta-analysis had been performed. These categories include the LE synthesis (without a meta-analysis) and living meta-analysis (with a meta-analysis conducted).

Second, the criteria for inclusion in this review are studies that use AI or semiautomated tools in the following phases of LE synthesis: (1) database searching and eligibility assessment, (2) data extraction or collection and risk of bias assessment, (3) synthesis and analysis, or (4) publication update [6]. The LE syntheses from any field were included. In addition, studies that developed AI or semiautomated tools for LE synthesis were also included. Textbox 1 provides further details.

Textbox 1. **Inclusion and exclusion criteria for the study.**

Inclusion criteria

- The studies using artificial intelligence (AI) or semiautomated tools in the following phases of living evidence (LE) synthesis: (1) database searching and eligibility assessment, (2) data extraction or collection and risk of bias assessment, (3) synthesis and analysis, or (4) publication update. A study can be any type of LE synthesis in any field, including but not limited to all scientific journals in the social sciences.
- Studies that developed AI or semiautomated tools for LE synthesis.

Exclusion criteria

- Studies that did not document the use of AI or semiautomated tools in LE synthesis.
- Protocol, commentaries, editorials, letters to the editor, and updating studies.

We excluded studies that did not document the use of AI or semiautomated tools in LE synthesis. In addition, protocols, commentaries, editorials, letters to the editor, and updating studies were also excluded, as shown in Textbox 1.

Third, AI tools are characterized by autonomous learning and end-to-end decision-making. They enable the independent execution of data collection, feature extraction, model training, and inference and generate output results without any human intervention. However, semiautomated tools incorporate human review or decision support at critical stages, using a “machine assistance and human oversight” collaborative paradigm [18,19]. Textbox 2 shows the types of AI or semiautomated tools, where AI or semiautomated tools were categorized by the application phases. First, the first segment of the AI or semiautomated tools for each phase is sourced from Bendersky et al [13]. Second, the subsequent segment is derived from the work of Khalil et al [20]. Third, for the final segment, AI or semiautomated tools were identified and summarized from relevant studies using a manual search. The AI techniques based on LLMs, such as the generative pretrained transformer, were also included.

Textbox 2. **Artificial intelligence (AI) or semiautomated tools used in the 4 phases of living evidence (LE) synthesis.**

Phase 1. Database searching and eligibility assessment

- Segment 1.1: Automatic, continuous database search with push notification, database aggregators (such as HDAS, Epistemonikos), notification from clinical trial registries, randomized clinical trial classifier, text mining technologies, and automatic retrieval of full-text papers
- Segment 1.2: RCT tagger, LitSuggest, Evidence mapping tool, SRA-Polyglot Search Translator, QuickClinical, HDAS, ROBOTsearch, SRA-word, frequency analyzer, The Search Refiner, Sherlock, SRA De-duplicate, Distiller, R package-rev tools, Rayyan, EPPI-reviewer, Abstrackr, SRA helper, LibSVM classifier, Bibot, Active Screener, RobotAnalyst, Swift-Review, Evidence Pipeline, JBI Sumari, EndNote, SARA, eSuRFr, ParsCit, and Citation searcher
- Segment 1.3: Natural language processing–assisted abstract screening tool, automatic text classifiers supported by deep learning–based language models, machine learning classifiers, Cochrane Crowd, Living Interactive Evidence (LIvE) synthesis platform, Cochrane RCT classifier, OpenAlex, Risklick AI, Bayesian classifier, Generative Pretrained Transformer models, and RobotReviewer LIVE

Phase 2. Data extraction or collection and risk of bias assessment

- Segment 2.1: Machine learning information-extraction systems, automated structured data extraction tools for PDFs, machine learning–assisted RoB tool, data repositories, and linked data
- Segment 2.2: RobotReviewer, DistelleR, JBI Sumari, in-house data extraction tool written in R, statistical package R, ExaCT, Revman, Raptor, ContentMine, Graph2Data, and Evidence mapping tool
- Segment 2.3: BioMart, MetaInsight COVID-19, LIvE synthesis platform, Open Science Framework (OSF), PsychOpen CAMA, and Generative Pretrained Transformer models

Phase 3. Synthesis and analysis

- Segment 3.1: Structured data extraction tools, which automatically provide data in a suitable format for statistical analysis; continuous analysis updating based on availability of structured extracted data; and statistical surveillance of key analysis results, with threshold set for potential conclusion change
- Segment 3.2: MetaPreg, MetaXL, NetMetaXL, Meta-analyst, Webplotdigitizer, Evidence mapping tool, PRISMA flow diagram generator, Evidence mapping tool, R package-rev tools
- Segment 3.3: Risklick AI, Web Source Processing Pipeline, LIvE synthesis platform, and generative pretrained transformer models

Phase 4. Publication update

- Segment 4.1: Templated reporting of some report items, automatic text generation tools for synthesis and writing, automatization in the identification of changes between LSR versions for peer review, and editorial process (such as Archie)
- Segment 4.2: Trial2rev, RevManHAL, DistelleR, SRA replicant writer, SRA-RevMan Replicant, and JBI Sumari
- Segment 4.3: Generative pretrained transformer models

### Study Screening and Data Collection

Two reviewers independently screened the titles and abstracts of all selected studies, followed by a full-text review. Any disagreements regarding selection were resolved by a third researcher. Data were extracted using a predesigned Microsoft Excel sheet. Two reviewers independently extracted data from all included studies, including information such as title, first author, journal, year of publication, LE synthesis type, types of tool or technology, types of AI or semiautomated tools, phases of LE synthesis, outcomes, and so forth. Any disagreements were resolved by a third researcher. During data extraction, representative outcomes (such as means or ranges) were prioritized for synthesis, with the range of values considered subsequently when outcomes were similarly representative.

### Methodological Quality Assessment

Given the lack of a standardized tool for assessing the methodological quality of AI-related studies, the 24 studies were categorized into 3 types by methodological characteristics and primary objective (diagnostic test, tool development, or—when neither applied—a general synthesis) and assessed for methodological quality using the modified version of the Quality Assessment of Diagnostic Accuracy Studies version 2 (QUADAS-2) tool, Joanna Briggs Institute (JBI) Critical Appraisal Checklist for Textual Evidence: Narrative, and AMSTAR 2 tool. First, 10 studies were assessed with the modified version of the QUADAS-2 tool: these studies specifically assessed the application of AI in the database searching and eligibility assessment phase, which aligns with a diagnostic test accuracy (DTA) framework. We adopted the modified version of the QUADAS-2 proposed by Rashid et al [21-23]. As QUADAS-2 is designed for DTA research contexts, this framework was only applicable to those studies where one of the objectives included the application of AI in the database searching and eligibility assessment phase [21,24,25]. The core elements of QUADAS-2 were revised to adapt it to AI-related research scenarios, as follows: “patient” was replaced with “study,” “index test” with “AI,” “reference standard” with “comparator,” and “case-control design” with “DTA framework.” We also constructed a 2×2 table, categorizing studies into “included” or “excluded” based on both “AI screening results” and “reference/original systematic review (SR) screening results,” with counts denoted as a, b, c, and d, respectively. The details of the modified QUADAS-2 are provided in Table S2 in Multimedia Appendix 1. Second, 5 studies, which specifically developed AI or semiautomated tools for LE synthesis without DTA-related accuracy evaluation and were not designed as LE synthesis themselves, were assessed using the JBI Critical Appraisal Checklist for Textual Evidence: Narrative [26]. Third, 9 studies, which were designed as LE syntheses without DTA-related accuracy evaluation and not primarily focused on AI or semiautomated tool development (or tool development was only an auxiliary means), were assessed using the AMSTAR 2 tool [27,28]. The details are shown in Tables S3 and S4 in Multimedia Appendix 1. All of the included studies were evaluated independently by 2 reviewers (RL and ZY), and disagreement was resolved by a third reviewer (ZL). The LE synthesis did not involve a statistical combination of results (meta-analysis), as its aims were to document the phases of LE synthesis where AI is used and to investigate whether AI improves the efficiency, accuracy, or utility of LE synthesis. Therefore, several systematic review procedures—including sensitivity analyses, reporting bias assessment, certainty assessment, and investigations of heterogeneity—were not used.

### Data Analysis

**Figure 1.**
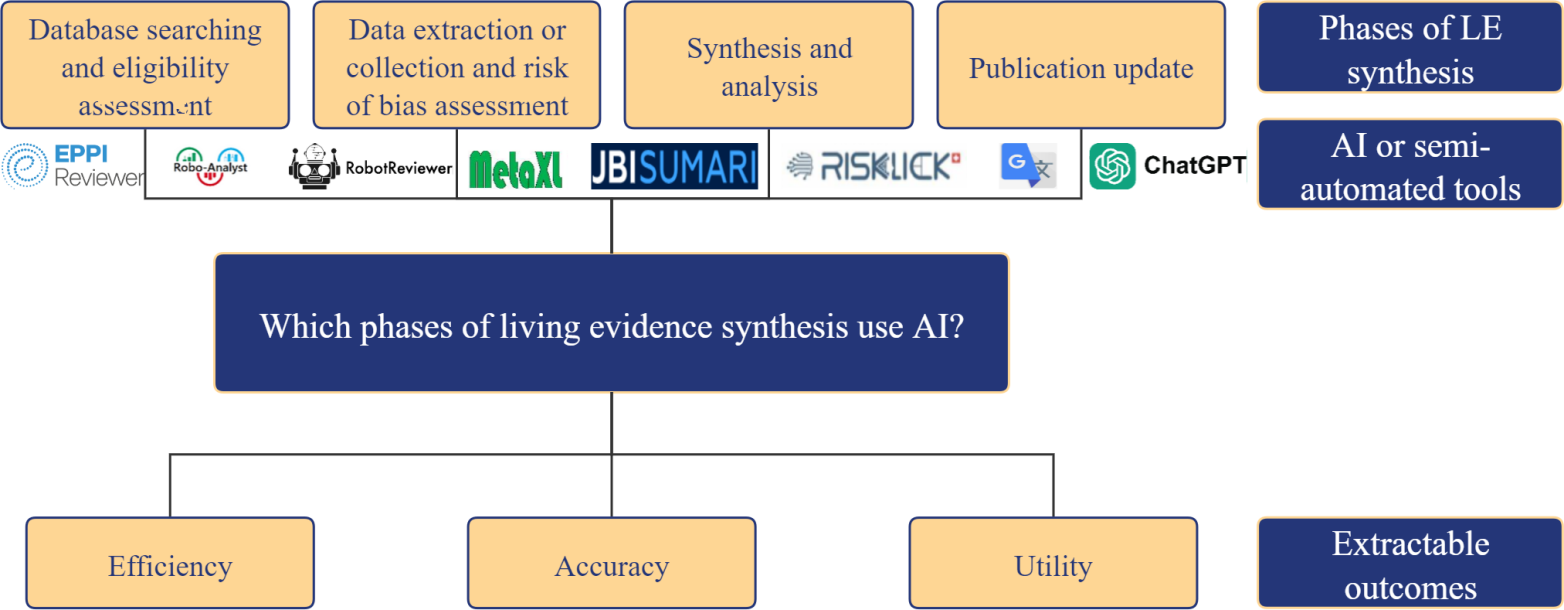
Road map for the use of artificial intelligence (AI): applications and extractable clinical outcomes across 4 phases of living evidence synthesis. LE: living evidence.

This review conducted 3 complementary analyses, as shown in Figure 1.

#### Analysis 1: Phases of LE Synthesis Utilizing AI or Semiautomated Tools

We analyzed the prevalence and distribution of AI or semiautomated tools across 4 phases of LE synthesis. Phase 1 is database searching and eligibility assessment. This process includes going through the databases, retrieving the results, importing them into the citation management software, removing any duplicate results, and assessing their eligibility individually. Phase 2 is data extraction or collection and risk of bias assessment; once the eligibility of studies has been verified and they have been included in the review process, it becomes crucial to systematically extract and collect information about their main characteristics and results. Additionally, it is very important to assess the risk of bias associated with the conduct and methodology used in the studies. In phase 3—synthesis and analysis—the data that have been assessed to conform to the criteria are integrated, and the data are analyzed. In phase 4—publication update—after going through the aforementioned phases 1-3, sections of a review are generated based on their results, and conclusions are updated.

#### Analysis 2: AI or Semiautomated Tools Used in LE Synthesis

First, the types of AI or semiautomated tools applied in each LE synthesis phase were investigated. Second, the frequency of AI or semiautomated tools applied in the LE synthesis was analyzed.

#### Analysis 3: Primary Outcomes Investigating AI or Semiautomated Tools in LE Synthesis

The impact of applied AI or semiautomated tools in LE synthesis was analyzed across 3 outcomes [29]. First, efficiency, defined as the relationship between the time required to complete a workload and the workload itself, was evaluated to determine whether either the duration or workload was reduced with the use of AI or semiautomated tools. This outcome may be described as time reduction, workload reduction, and conflict rates with and without the tool.

Second, accuracy is used to assess performance with and without AI or semiautomated tools. It may be described as accuracy, recall, precision, *F*_1_-score, area under the receiver operating characteristic curve, number needed to read, and study relevance. In addition, we calculated the overall mean recall and mean *F*_1_-score using the following formula:


$$\overset{ˉ}{M}=\frac{1}{N}\;\sum_{i=1}^{N}M_{i}$$

where $M_{i}$ is the representative value for study i, defined as the reported single value, if provided, or the midpoint of the reported range [L, U], calculated as (L+U)/2, if a range was provided. N is the number of studies reporting that metric [30,31].

Third, utility is used to assess whether user decisions align with those of AI or semiautomated tools, including user consistency, user satisfaction, perceived ease of use, and study quality.

## Results

### Search Results

**Figure 2.**
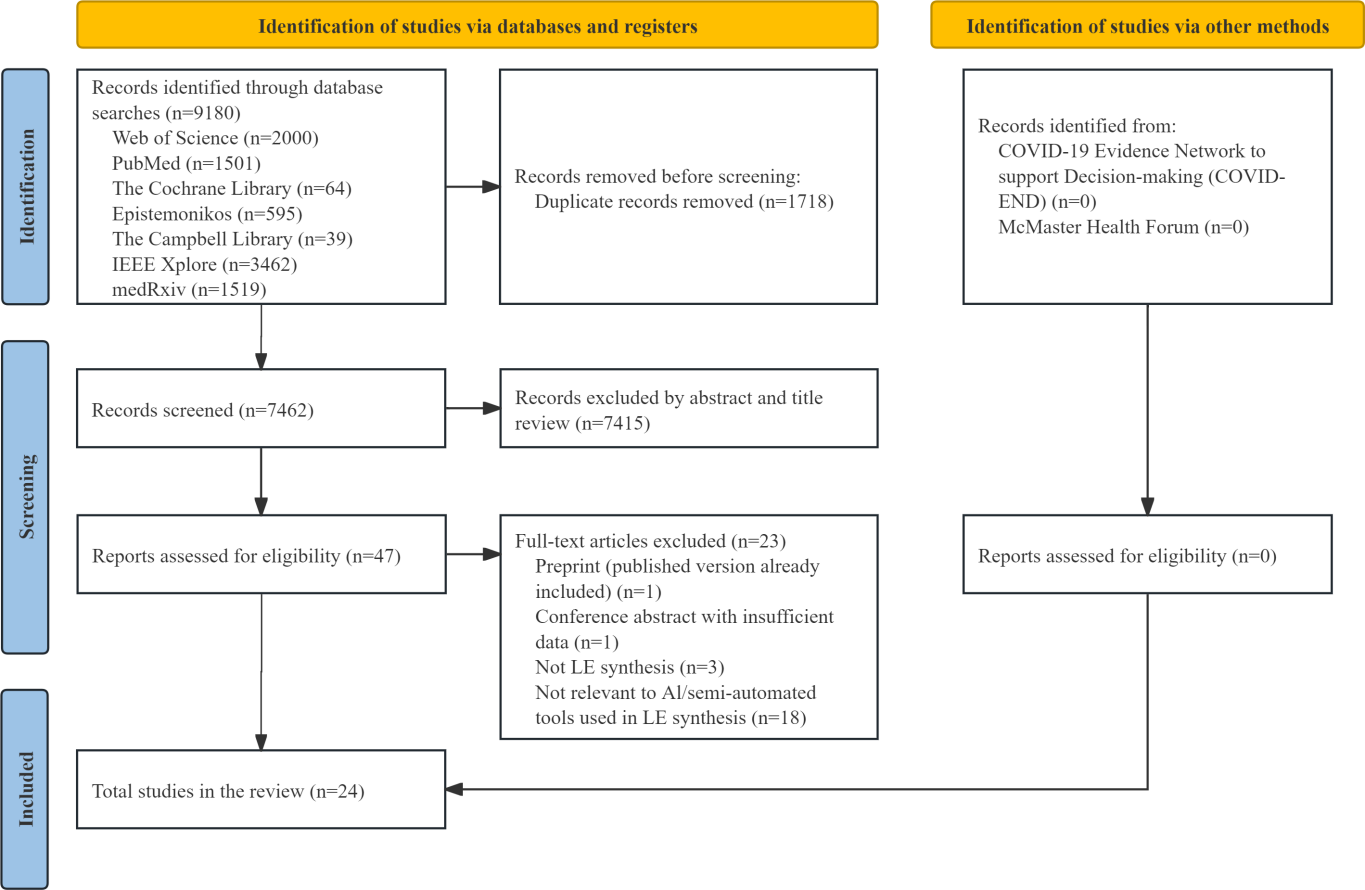
Database search flow diagram. LE: living evidence.

Out of 9180 studies, 24 studies applied AI or semiautomated tools in LE synthesis, including 17 LE syntheses (4 developing tools) and 7 living meta-analyses (3 developing tools), as shown in Figure 2 [29,32-54]. In addition, 8 studies exclusively applied AI tools in LE synthesis, 11 studies exclusively applied semiautomated tools, and 5 studies utilized both AI and semiautomated tools. The basic characteristics of the included studies are shown in Table S5 in Multimedia Appendix 1. The details of the studies excluded at the full-text eligibility stage with reasons are shown in Table S6 in Multimedia Appendix 1 [5,9,55-75].

### Methodological Quality of Included Studies

We conducted a methodological quality assessment of 10 studies using a revised QUADAS-2 tool within the DTA framework [29,32,35,36,42-44,51,52,54]. All studies were assessed as low-risk in the “Study selection,” “Index test (AI),” and “Reference (comparator)” domains. While none of the studies specified the time interval between the task execution of AI and comparator-based analysis, all were determined as low-risk in the “Flow and timing” domain. Additionally, we did not identify any applicability concerns, as all studies were classified as low-risk in the “Applicability” domain (Table 1). Five studies were subjected to methodological quality assessment using the JBI Critical Appraisal Checklist for Textual Evidence: Narrative [41,46,48-50]. Four studies obtained a score of 5/6, with a narrative appraisal of “Exclude” owing to failure to meet the narrative classification criterion [41,46,48,49]. One study achieved a full score of 6/6 and was thus appraised as “Include” (Table S7 in Multimedia Appendix 1) [50]. In addition, we conducted a methodological quality assessment of 9 studies using AMSTAR 2 [33,34,37-40,45,47,53]. The methodological quality scores of the included studies ranged from 11 to 15. Overall, the methodological quality of eight studies [34,37-40,45,47,53] was rated as moderate, while only 1 study [33] was rated as low in methodological quality. The most common limitation was that the authors failed to provide a list of excluded studies (Table S8 in Multimedia Appendix 1).

**Table 1.** Summary of modified Quality Assessment of Diagnostic Accuracy Studies version 2 (QUADAS-2) assessments for studies using artificial intelligence (AI) or semiautomated tools in the database searching and eligibility phase of the living evidence (LE) synthesis process.

Author, year	Risk of bias				Applicability concern		
	Study selection	Index test (AI)	Reference (comparator)	Flow and timing	Study selection	Index test (AI)	Reference (comparator)
Knafou et al [32] (2023)	Low	Low	Low	Low	Low	Low	Low
Perlman-Arrow et al [29] (2023)	Low	Low	Low	Low	Low	Low	Low
Chou et al [35] (2020)	Low	Low	Low	Low	Low	Low	Low
Kamso et al [36] (2023)	Low	Low	Low	Low	Low	Low	Low
Marshall et al [42] (2023)	Low	Low	Low	Low	Low	Low	Low
Haas et al [43] (2021)	Low	Low	Low	Low	Low	Low	Low
Vaghela et al [44] (2021)	Low	Low	Low	Low	Low	Low	Low
Shemilt et al [51] (2024)	Low	Low	Low	Low	Low	Low	Low
Le-Khac et al [52] (2024)	Low	Low	Low	Low	Low	Low	Low
Hair et al [54] (2024)	Low	Low	Low	Low	Low	Low	Low

### Types and Frequency of AI or Semiautomated Tools in LE Synthesis

A total of 34 AI or semiautomated tools were involved, including 12 (35.3%) AI tools and 22 (64.7%) semiautomated tools, as shown in Multimedia Appendix 2. The most frequently used AI or semiautomated tools were machine learning classifiers (n=5), followed by the Living Interactive Evidence (LIvE) synthesis platform (n=3), AD-SOLES (n=2), Covidence (n=2), and MAGICapp (n=2).

### Phases of AI or Semiautomated Tools Application in LE Synthesis

There were 18 AI or semiautomated tools for database searching and eligibility assessment, 20 for data extraction or collection and risk of bias assessment, and 10 for synthesis and analysis. However, only 1 AI tool was used for publication updates. Out of all the tools, RobotReviewer LIVE can be used for all phases of LE synthesis, as shown in Textbox 3.

Textbox 3. **Types of artificial intelligence (AI) or semiautomated tools applications in the 4 phases of living evidence (LE) synthesis.**

Phase 1. Database searching and eligibility assessment

- LIvE platform, automatic text classifiers, machine learning ensemble classifier, Natural language processing–assisted abstract screening tool, machine learning classifiers, machine learning, PICO annotators, STAR tool, AD-SOLES, Covidence, rcrossref, openalexR, RISmed, RobotReviewer LIVE, Risklick AI, metaCOVID application, supervised text classification models, and text mining techniques

Phase 2. Data extraction or collection and risk of bias assessment

- LIvE platform, web-based interactive app, open-source living systematic review application, Covidence, AD-SOLES, Google Refine tool, script, REDASA, RobotReviewer LIVE, Risklick AI, Metainsight COVID-19, metaCOVID application, information extraction techniques, EndNote, semiautomated model, supervised text classification models, text mining techniques, GPT-4-turbo, Claude-3-Opus, and EPPI-Reviewer

Phase 3. Synthesis and analysis

- LIvE platform, MAGICapp, Trial sequential analysis (TSA) software, AD-SOLES, ODDPub, RobotReviewer LIVE, script, Metainsight COVID-19, metaCOVID application, and Dynameta

Phase 4. Publication update

- RobotReviewer LIVE

### Impact of AI or Semiautomated Tools on LE Synthesis

#### Overview

A total of 10 (41.7%) studies reported on the impact of AI or semiautomated tools on LE synthesis in terms of efficiency, accuracy, or utility in the database searching and eligibility phase or the data extraction or collection and risk of bias assessment phase. Table 2 provides a description of the outcome metrics in the included studies.

**Table 2.** Summary of the indicator terms for outcome metrics in the included studies.

Metrics	Explanation
Efficiency	
Time	AI^a^ or semiautomated tools were used to save time. Only 2 (8.3%) studies reported on time saving [29,35]. Specifically, Perlman-Arrow et al [29] reported a 45.9% reduction in screening time per abstract in the database searching and eligibility phase. Chou et al [35] estimated the time saving ranged from 2.0 to 13.2 hours in the database searching and eligibility phase.
Workload	Two (8.3%) studies reported on workload metrics related to the use of AI or semiautomated tools [29,42]. Perlman-Arrow et al [29] reported that the semiautomated tool completed 68% of the workload in the database searching and eligibility phase. Marshall et al [42] found that manual screening had an efficiency rate of 23% in obtaining 31 abstracts, whereas AI achieved a rate of 55%, demonstrating an efficiency improvement of approximately 140% in the database searching and eligibility phase.
Conflict rates with and without the tool	The efficiency of abstract screening decreases as the number of conflicting votes increases [29]. Perlman-Arrow et al [29] reported a reduction in conflict rates from 8.32% to 3.64% with the use of semiautomated tool in the database searching and eligibility phase.
Accuracy^b^	
Precision	Precision refers to the ratio of accurately categorized documents among all the documents that the model assigns to a particular class [32]. Eight (33.3%) studies reported on precision [29,32,35,42,43,51,53,54]. Khan et al [53] reported a precision rate of even 100% using AI in the data extraction or collection and risk of bias assessment phase. Perlman-Arrow et al [29] and Haas et al [43] reported precision rates of 92.10% and 96.07%, respectively, using AI or semiautomated tools in the database searching and eligibility phase. Hair et al [54] reported that the average precision rate using AI is about 84.5% in the database searching and eligibility phase. Shemilt et al [51] reported a precision rate of 50%‐86% using AI in the database searching and eligibility phase. Marshall et al [42] reported a precision rate of 55% using AI in the database searching and eligibility phase. Knafou et al [32] reported a precision rate of only 29.69% using AI in the database searching and eligibility phase. However, Chou et al [35] reported a precision rate of only 0.2%‐8% using AI in the database searching and eligibility phase.
Recall^c^	Recall (also known as sensitivity) refers to the fraction of positive documents that have been accurately identified among all documents for the specified class [32]. Nine (37.5%) studies reported on recall [29,32,35,36,42,43,51,53,54]. All studies reported recall rates in excess of 87%. The average value was about 96.24%. Perlman-Arrow et al [29], Chou et al [35], and Marshall et al [42] found recall rates of even 100% using AI or semiautomated tools in the database searching and eligibility phase. Knafou et al [32], Haas et al [43], and Kamso et al [36] reported a recall rate of 89%, 99.25% and 99.3%, respectively, using AI in the database searching and eligibility phase. Shemilt et al [51] reported a recall rate of 94%‐99% using AI in the database searching and eligibility phase. Khan et al [53] reported a recall rate of 92%‐96% using AI in the data extraction or collection and risk of bias assessment phase. Hair et al [54] reported that the average sensitivity rate using AI is about 95.1% in the database searching and eligibility phase.
*F*_1_-score^c^	*F*_1_-score refers to the balanced harmonic average between the model precision and recall [32]. Six (25%) studies reported on *F*_1_-score [29,32,43,52-54]. All studies reported *F*_1_-score between 80.47% and 99% after using AI. The average value was about 92.17%. Knafou et al [32], Perlman-Arrow et al [29], and Haas et al [43] reported an *F*_1_-score of 89.2%, 92.6%, and 97.59%, respectively, using AI or semiautomated tools in the database searching and eligibility phase. Le-Khac et al [52] reported an *F*_1_-score of 87% using AI in the data extraction or collection and risk of bias assessment phase. Khan et al [53] reported *F*_1_-scores between 96% and 98% after using AI in the data extraction or collection and risk of bias assessment phase. Hair et al [54] reported that the average *F*_1_-score using AI is about 89.6% in the database searching and eligibility phase.
Area under the receiver operating characteristic curve (AUC-ROC)	AUC-ROC calculates the area under the curve between the true positive rate and the false positive rate [32]. Knafou et al [32] reported higher AUC-ROC performance using AI in the database searching and eligibility phase and had an AUC-ROC performance of 94.25%‐94.77%.
Number needed to read (NNR)	NNR refers to the total number of literature considered within the search divided by the number of literature included from the search [35]. Only 2 (8.3%) studies reported on NNR [29,35]. Perlman-Arrow et al [29] reported an NNR between 1.086 and 1.125 after using a semiautomated tool in the database searching and eligibility phase. Chou et al [35] reported an NNR between 15 and 100 after using AI in the database searching and eligibility phase.
Article relevance	Vaghela et al [44] reported on studies included after searching using AI, and 50.49% were considered relevant to the query in the database searching and eligibility phase.
Utility	
User satisfaction	Perlman-Arrow et al [29] reported that the average satisfaction of users with the tool reached 4.2/5 in the database searching and eligibility phase.
Consistency	Kamso et al [36] reported that consistency in the use of AI between 2 reviewers was assessed using percentage agreement and Kappa scores, revealing a range of percentage agreement from 79.0% to 96.0%, and a variation in Kappa scores from moderate (0.40) to substantial (0.63) in the database searching and eligibility phase.
Article quality	Vaghela et al [44] reported that 64.53% of the included studies possess reliable quality in the database searching and eligibility phase.

a AI: artificial intelligence.

b Kamso et al [36] achieved an accuracy ranging from 75.9% to 96.9% in research classification using AI in the database searching and eligibility phase. Khan et al [53] reported that the collaborative large language models’ accuracy, based on concordant responses in the prompt set, reached 99% in the data extraction or collection and risk of bias assessment phase.

c The overall mean recall (96.24%) and *F*_1_-score (92.17%) are the simple averages of study‑level values from Table S5 in Multimedia Appendix 1. For studies reporting a range, the midpoint was used as the study‑level value.

#### Efficiency Enhancements Through AI or Semiautomated Tools in LE Synthesis

Three studies showed improved efficiency in the database searching and eligibility phase in terms of 3 indicator terms. A total of 2 (8.3%) studies [29,35] reported on time saving with AI or semiautomated tools, 2 (8.3%) studies [29,42] reported on workload metrics related to the use of AI or semiautomated tools, and 1 study [29] reported a reduction in conflict rates with the use of semiautomated tool, which consequently increases the efficiency.

#### Accuracy Improvements With AI or Semiautomated Tools in LE Synthesis

A total of 9 and 6 studies that applied AI or semiautomated tools in LE synthesis reported a mean recall rate and a mean *F*_1_-score of 96.24% and 92.17%, respectively. While Khan et al [53] reported a precision rate of even 100% achieved using AI in the data extraction or collection and risk of bias assessment phase. However, in 7 studies, the reported precision rates varied significantly, ranging from 0.2% to 96.07% in the database searching and eligibility phase.

#### Utility of AI or Semiautomated Tools in LE Synthesis

Three studies reported on the utility of AI or semiautomated tools in the database searching and eligibility phase of LE synthesis, including user satisfaction, consistency, and study quality. Consistency in the use of AI between 2 reviewers was assessed using percentage agreement and Kappa scores [36].

## Discussion

### Principal Findings

AI or semiautomated tools are actively used to facilitate the process of LE synthesis. We conducted this review to identify the phases of LE synthesis that use AI and explore whether AI can improve the efficiency, accuracy, or utility of LE synthesis.

AI or semiautomated tools have been increasingly used in LE synthesis, particularly in living systematic review. This review discovered that AI or semiautomated tools are most commonly used for data extraction or collection and risk of bias assessment. However, only a few studies have addressed the use of AI or semiautomated systems for publication updates, highlighting the need for further development in this phase.

Diverse types of AI or semiautomated tools were identified in this study. These include the LIvE synthesis platform, AD-SOLES, metaCOVID application, and RobotReviewer LIVE, which are utilized in multiple phases of LE synthesis, indicating their versatility and potential for wider adoption [37,39,40,42,47,54]. The most frequently used AI or semiautomated tools were machine learning classifiers, the LIvE synthesis platform, Covidence, AD-SOLES, and MAGICapp. Furthermore, the rapid rise of AI tools involving LLM types, such as GPT-4-turbo and Claude-3-Opus, has led to their use in LE synthesis. These tools can be suitable for application in multiple or even all phases of LE synthesis, especially in the publication update phase. The application of LLMs to further enhance the efficiency, accuracy, and utility of LE synthesis remains a key focus for researchers and practitioners.

Governments worldwide, particularly those in leading AI nations such as China, the United States, Germany, the United Kingdom, France, and Canada, are especially emphasizing the transformative impact of AI on research and decision-making processes [76,77]. Funding from various sources, including the Economic and Social Research Council, reflects a strong financial commitment to advancing AI technologies in evidence synthesis. Furthermore, a growing number of AI guidance and organizations are emerging to embrace the opportunity that AI has taken in producing LE synthesis. For example, Responsible AI in Evidence SynthEsis has provided recommendations for the main roles of responsible AI in the evidence synthesis ecosystem that are involved in responsible AI use [78]. Furthermore, organizations such as ALIVE aim to improve societal outcomes by producing and utilizing timely, trustworthy, and affordable evidence.

Challenges remain in the application of AI in LE synthesis. Machine learning classifiers suffer from low precision and varying efficiency across different topics [35]. As an example, RobotReviewer LIVE faces challenges in performance variability for complex reviews, limited study types, and data source constraints [42]. Therefore, further research aimed at enhancing the adaptability and stability of AI across various research areas is urgently needed. In addition, ethical issues, data protection measures, and transparency in AI-driven LE synthesis are also key challenges that need to be addressed [79]. At the ethical level, AI is prone to selection bias due to the skewness of its training data, which impairs the inclusivity of evidence, and the mechanism of responsibility attribution remains unclear [80]. Data protection is another area that faces challenges, as research data required for AI training often contain sensitive information, and existing anonymization technologies cannot fully avoid the risk of privacy breaches [81]. Cost considerations in the implementation of AI tools, including initial investment, ongoing operational costs, training expenses, and requirements for hardware and software resources also constitute a significant issue [82].

Policymaking involves judgment, making it more of an art than a science, whereas science is primarily driven by evidence and shapes evidence-informed policymaking [83]. Study has indicated that relying solely on systematic reviews for policymaking is far from sufficient; instead, policymakers need to obtain a more diverse range of synthesized evidence to underpin decision-making [84]. The LE synthesis, especially by incorporating AI into evidence production, can deliver updated evidence to facilitate evidence-informed policymaking. AI could revolutionize policymaking by facilitating ongoing assessments, ensuring that the policies remain aligned with the latest evidence and evolve in response to new information as it emerges [2,5,85]. Furthermore, AI enables policymakers to continuously monitor and assess policies throughout their lifecycle, which allows adaptation to shifting circumstances and evolving societal needs in real time [86]. Furthermore, the advancement of AI capabilities, particularly through LLMs, adds a deeper analytical layer; LLMs can provide nuanced insights and help predict future research directions relevant to policymaking [87]. The application of AI in LE synthesis could transform policy decision-making, advancing policy formulation for policymakers.

Recent advances in AI provide researchers with new transformative capabilities [79]. Van Dijk et al [88] indicated that AI tools are a promising innovation in the current practice of systematic evaluation, and researchers have reported positive experiences with these tools. The use of AI enhances efficiency by significantly reducing researchers’ time and workload [2,89]. Manion et al [90] indicated that natural language processing could enhance accuracy and reduce errors through a “human-in-the-loop” approach. The application of AI in LE synthesis has considerably benefited researchers, significantly enhancing their research capabilities.

This LE synthesis will retain its living mode beyond the present publication, consistent with the methodology. This decision is based on two key considerations: (1) the predefined retirement triggers have not been triggered and (2) the Safe and Responsible Use of AI Working Group (Working Group 3) and the Methods & Process Innovation Working Group (Working Group 4) of the Evidence Synthesis Infrastructure Collaborative will benefit from the continuous updates from this LE synthesis to support their future research initiatives [91-94].

### Future Research Directions

In the above discussion, we have suggested the advancement of future work across multiple dimensions. From a technical point of view, efforts are needed to address limitations of existing AI tools, such as inadequate precision and poor adaptability, while deepening research into the LLM applications in the publication update phase of LE synthesis. In the realm of ethics and data governance, it is essential to establish responsibility attribution mechanisms and cross-regulatory data governance frameworks, as well as enhance evidence inclusivity and mitigate privacy risks through algorithmic optimization. Methodologically, we recommend the establishment of a standardized evaluation system for AI applications and refining research design and quality assessment protocols to strengthen the evidence base.

### Strengths and Limitations

The strengths of this review include the following: (1) it systematically analyzes the types of AI and semiautomated tools used across the 4 phases of LE synthesis and (2) it provides insights into the opportunities and challenges of using AI or semiautomated tools in LE synthesis regarding efficiency, accuracy, and utility. However, this review still has a few limitations. First, study screening was based on whether the studies reported on the tools used in LE synthesis. Second, studies that did not document the use of AI or semiautomated tools in LE synthesis were excluded from this review, which may introduce bias. Third, the focus of our search strategy on “living evidence” terminology may have excluded studies describing AI tools for review updates that used different terminology.

### Conclusion

Researchers are actively utilizing various AI and semiautomated tools in LE synthesis, primarily for data extraction or collection and risk of bias assessment, while their application in updating publications remains limited. The use of AI or semiautomated tools in LE synthesis improves efficiency in the database searching and eligibility phase and accuracy in the database searching and eligibility phase, as well as in the data extraction or collection and risk of bias assessment phase. The AI or semiautomated tools demonstrate high accuracy, recall, and *F*_1_-scores, while precision varies across tools. AI or semiautomated tools also demonstrate good performance in terms of utility in the database searching and eligibility phase.

## Supplementary material

10.2196/76130 Multimedia Appendix 1 Search strategies, included and excluded study information, and methodological quality assessment methods and results.

10.2196/76130 Multimedia Appendix 2 Frequency of artificial intelligence (AI) or semiautomated tools use.

10.2196/76130 Checklist 1 PRISMA-LSR checklist.

## Abbreviations

AI: artificial intelligence
DTA: diagnostic test accuracy
JBI: Joanna Briggs Institute
LE: living evidence
LIvE: Living Interactive Evidence
LLM: large language model
PRISMA-LSR: Preferred Reporting Items for Systematic Reviews and Meta-Analyses 2020 statement for living systematic reviews
QUADAS-2: Quality Assessment of Diagnostic Accuracy Studies version 2
