# Supplementary material for: The Phases of Living Evidence Synthesis Using AI: Living Evidence Synthesis (Version 1)
Source: J Med Internet Res. 2026 Jan 27;28:e76130. doi: 10.2196/76130 (PMC12842881; doi:10.2196/76130)
Supplement: Multimedia Appendix 1 [file jmir-v28-e76130-s001.docx]

Table S1. Search strategies for all filtered databases.

| **No.** | **Search strategy** | **Results** |
| --- | --- | --- |
| **PubMed** | | |
| #1 | "continuous updat*"[Title/Abstract] OR "continually updat*"[Title/Abstract] OR "constant updat*"[Title/Abstract] | 1,272 |
| #2 | review*[Title/Abstract] OR "meta-analys*"[Title/Abstract] OR evidence[Title/Abstract] OR synthes*[Title/Abstract] OR guideline*[Title/Abstract] | 6,559,553 |
| #3 | "Meta-Analysis" [Publication Type] OR "Meta-Analysis as Topic"[Mesh] OR "Network Meta-Analysis" [Publication Type] OR "Network Meta-Analysis as Topic"[Mesh] OR "Review" [Publication Type] OR "Review Literature as Topic"[Mesh] OR "Guideline" [Publication Type] OR "Guidelines as Topic"[Mesh] | 3,902,632 |
| #4 | #2 OR #3 | 8,105,434 |
| #5 | #1 AND #4 | 635 |
| #6 | "living systematic review*"[Title/Abstract] OR "living meta-analys*"[Title/Abstract] OR "living rapid review*"[Title/Abstract] OR "living review*"[Title/Abstract] OR "living evidence"[Title/Abstract] OR "living guideline*"[Title/Abstract] | 849 |
| #7 | "continuous itera*"[Title/Abstract] OR "constant itera*"[Title/Abstract] | 57 |
| #8 | #6 OR #7 | 906 |
| #9 | #5 OR #8 | 1,501 |
| **Web of Science** | | |
| #1 | TS=(“continuous updat*” OR “continually updat*” OR “constant updat*”) | 2,968 |
| #2 | TS=(review* OR “meta-analys*” OR evidence OR synthes* OR guideline*) | 10,308,961 |
| #3 | #1 AND #2 | 754 |
| #4 | TS=(“living systematic review*” OR “living systematic synthes*” OR “living meta-analys*” OR “living rapid review*” OR “living rapid evidence” OR “living review*” OR “living evidence” OR “living guideline*”) OR (“continuous itera*” OR “continually itera*” OR “constant itera*”) | 1,280 |
| #5 | #3 OR #4 | 2,000 |
| **The Cochrane Library** | | |
| #1 | (continuous NEXT updat*) OR (continually NEXT updat*) OR (constant NEXT updat*):ti,ab,kw | 96 |
| #2 | (review*) OR (meta-analys*) OR (evidence) OR (synthes*) OR (guideline*):ti,ab,kw | 266,569 |
| #3 | #1 AND #2 | 40 |
| #4 | ("living systematic" NEXT review*) OR ("living systematic" NEXT synthes*) OR (living NEXT meta-analys*) OR ("living rapid" NEXT review*) OR ("living rapid evidence"):ti,ab,kw | 5 |
| #5 | (living NEXT review*) OR ("living evidence") OR (living NEXT guideline*) OR (continuous NEXT itera*) OR (continually NEXT itera*) OR (constant NEXT itera*):ti,ab,kw | 20 |
| #6 | #4 OR #5 | 25 |
| #7 | #3 OR #6 | 64 |
| **Epistemonikos** | | |
| #1 | (title:((("continuous updat*" OR "continually updat*" OR "constant updat*") AND (review* OR "meta-analys*" OR evidence OR synthes* OR guideline*)) OR (("living systematic review*" OR "living systematic synthes*" OR "living meta-analys*" OR "living rapid review*" OR "living rapid evidence" OR "living review*" OR "living evidence" OR "living guideline*") OR ("continuous itera*" OR "continually itera*" OR "constant itera*"))) OR abstract:((("continuous updat*" OR "continually updat*" OR "constant updat*") AND (review* OR "meta-analys*" OR evidence OR synthes* OR guideline*)) OR (("living systematic review*" OR "living systematic synthes*" OR "living meta-analys*" OR "living rapid review*" OR "living rapid evidence" OR "living review*" OR "living evidence" OR "living guideline*") OR ("continuous itera*" OR "continually itera*" OR "constant itera*")))) | 595 |
| **IEEE Xplore** | | |
| #1 | (((("Abstract":review OR "Abstract":“meta-analys*” OR "Abstract":evidence OR "Abstract":synthesis OR "Abstract":guideline) AND ("Abstract":“Continuous updat*” OR "Abstract":“continually updat*” OR "Abstract":“constant updat*”)) OR (("Abstract":“living systematic review” OR "Abstract":“living systematic synthesis” OR "Abstract":“living meta-analys*” OR "Abstract":“living rapid review” OR "Abstract":“living rapid evidence” OR "Abstract":“living review” OR "Abstract":“living evidence” OR "Abstract":“living guideline” OR "Abstract":“continuous itera*” OR "Abstract":“continually itera*” OR "Abstract":“constant itera*”)))) | 3,462 |
| **The Campbell Library** | | |
| #1 | (("continuous updat*" OR "continually updat*" OR "constant updat*") AND (review* OR "meta-analys*" OR evidence OR synthes* OR guideline*)) OR (("living systematic review*" OR "living systematic synthes*" OR "living meta-analys*” OR "living rapid review*" OR "living rapid evidence" OR "living review*" OR "living evidence" OR "living guideline*") OR ("continuous itera*" OR "continually itera*" OR "constant itera*")) in Abstract | 39 |
| **medRxiv** | | |
| #1 | abstract or title "continuous update" (match all words) | 26 |
| #2 | abstract or title "continually update" (match all words) | 1 |
| #3 | abstract or title "constant update" (match all words) | 11 |
| #4 | abstract or title "living systematic review" (match all words) | 262 |
| #5 | abstract or title "living systematic synthesis" (match all words) | 62 |
| #6 | abstract or title "living meta-analysis" (match all words) | 129 |
| #7 | abstract or title "living rapid review" (match all words) | 121 |
| #8 | abstract or title "living rapid evidence" (match all words) | 92 |
| #9 | abstract or title "living review" (match all words) | 1,164 |
| #10 | abstract or title "living evidence" (match all words) | 653 |
| #11 | abstract or title "living guideline" (match all words) | 35 |
| #12 | #1 OR #2 OR #3 OR #4 OR #5 OR #6 OR #7 OR #8 OR #9 OR #10 OR #11 | 1,519 |

Table S2. Modifications made in QUADAS-2 for quality assessment of AI studies.

| **Domain** | **No** | **Questions** | **Grading** |
| --- | --- | --- | --- |
| Patient (Study) selection | 1 | Was a consecutive or random sample of patients enrolled (studies retrieved)? | Y/N/UC |
|  | 2 | Was a case-control design avoided? (Was a Diagnostic test accuracy framework adopted) | Y/N/UC |
|  | 3 | Did the study avoid inappropriate exclusions? | Y/N/UC |
|  | 4 | Could the selection of patients (studies) have introduced bias? Overall risk of bias in patient selection | L/H/UC |
|  | 5 | Applicability: Describe number of included patients (studies) (prior testing, presentation, intended use of index test and setting) | L/H/UC |
|  | 6 | Are there concerns that the included patients (studies) do not match the review question? Level of concern regarding applicability | Y/N/UC |
| Index test (AI) | 7 | Describe the index test (AI model) and how it was conducted and interpreted: | Description |
|  | 8 | Were the index test (AI model) results interpreted without knowledge of the results of the reference standard? | Y/N/UC |
|  | 9 | If a threshold was used, was it pre-specified? | Y/N/UC |
|  | 10 | Could the conduct or interpretation of the index test (AI model) have introduced bias? Concern on the risk of bias in the conduct or interpretation of the index test (AI model) | L/H/UC |
|  | 11 | Are there concerns that the index test (AI model), its conduct, or interpretation differ from the review question? Concern regarding applicability | L/H/UC |
| Reference (comparator) | 12 | Describe the reference standard (comparator) and how it was conducted and interpreted | Description |
|  | 13 | Is the reference standard (comparator) likely to correctly classify the target condition? | Y/N/UC |
|  | 14 | Were the reference standard (comparator) results interpreted without knowledge of the results of the index test? (AI model) | Y/N/UC |
|  | 15 | Could the reference standard (comparator), its conduct, or its interpretation have introduced bias? Level of concern | L/H/UC |
|  | 16 | Are there concerns that the target condition as defined by the reference standard (comparator) does not match the review question? Level of concern | L/H/UC |
| Flow and timing | 17 | Describe any patients who (studies that) did not receive the index test(s) (AI model (s)) and/or reference standard or who were excluded from the 2x2 table (refer to flow diagram) (if none or no statement, please state so) | Description |
|  | 18 | Describe the time interval between index test(s) (AI model (s)) and reference standard (comparator) (if no statement please put NS) | Description |
|  | 19 | Was there an appropriate interval between index test(s) (AI model (s)) and reference standard? | Y/N/UC |
|  | 20 | Did all patients receive a reference standard? Did all studies processed by the comparator? | Y/N/UC |
|  | 21 | Did all patients receive the same reference standard? Did all studies processed by the same comparator? | Y/N/UC |
|  | 22 | Were all patients (studies) included in the analysis? | Y/N/UC |
|  | 23 | Could the patient (study) flow have introduced bias? Risk of bias | L/H/UC |
|  | 24 | Please state here if you have any other comment |  |

Notes:1. The red text indicates the terms in the original QUADAS-2 tool, and the green text indicates the modifications made for this study. 2. AI: Artificial intelligence; L: Low, H: High; N: No; UC: Unclear; Y: Yes.

Table S3. The JBI Critical Appraisal Checklist for Textual Evidence: Narrative.

| **No** | **Questions** | **Assessment Options** |
| --- | --- | --- |
| 1 | Is the generator of the narrative a credible or appropriate source? | Yes/No/Unclear/Not applicable |
| 2 | Is the relationship between the text and its context explained? (where, when, who with, how) | Yes/No/Unclear/Not applicable |
| 3 | Does the narrative present the events using a logical sequence so the reader or listener can understand how it unfolds? | Yes/No/Unclear/Not applicable |
| 4 | Do you, as reader or listener of the narrative, arrive at similar conclusions to those drawn by the narrator? | Yes/No/Unclear/Not applicable |
| 5 | Do the conclusions flow from the narrative account? | Yes/No/Unclear/Not applicable |
| 6 | Do you consider this account to be a narrative? | Yes/No/Unclear/Not applicable |

Notes: Quality Grade and Criteria: After item-level appraisal, a score out of six is produced (such as 6/6, 5/6), and an overall judgment is made: “Include”: the article meets all of the Narrative‑type criteria and can be used as narrative evidence in the review; “Exclude”: the article fails to satisfy one or more critical Narrative-type criteria; therefore, it is excluded from the Narrative-type evidence pool; “Seek further information”: the information provided is insufficient to make a definitive judgment, and additional clarification is required before a final decision can be made.

Table S4. AMSTAR 2 for quality assessment of included studies.

| **No** | **Item Content** | **Assessment Options** |
| --- | --- | --- |
| 1 | Did the research questions and inclusion criteria for the review include the components of PICO? | Yes/Partial Yes/No |
| 2 | Did the report of the review contain an explicit statement that the review methods were established prior to the conduct of the review and did the report justify any significant deviations from the protocol? | Yes/Partial Yes/No |
| 3 | Did the review authors explain their selection of the study designs for inclusion in the review? | Yes/Partial Yes/No |
| 4 | Did the review authors use a comprehensive literature search strategy? | Yes/Partial Yes/No |
| 5 | Did the review authors perform study selection in duplicate? | Yes/Partial Yes/No |
| 6 | Did the review authors perform data extraction in duplicate? | Yes/Partial Yes/No |
| 7 | Did the review authors provide a list of excluded studies and justify the exclusions? | Yes/Partial Yes/No |
| 8 | Did the review authors describe the included studies in adequate detail? | Yes/Partial Yes/No |
| 9 | Did the review authors use a satisfactory technique for assessing the risk of bias (RoB) in individual studies that were included in the review? | Yes/Partial Yes/No |
| 10 | Did the review authors report on the sources of funding for the studies included in the review? | Yes/Partial Yes/No |
| 11 | If meta‑analysis was performed did the review authors use appropriate methods for statistical combination of results? | Yes/Partial Yes/No |
| 12 | If meta‑analysis was performed, did the review authors assess the potential impact of RoB in individual studies on the results of the meta‑analysis or other evidence synthesis? | Yes/Partial Yes/No |
| 13 | Did the review authors account for RoB in individual studies when interpreting/discussing the results of the review? | Yes/Partial Yes/No |
| 14 | Did the review authors provide a satisfactory explanation for, and discussion of, any heterogeneity observed in the results of the review? | Yes/Partial Yes/No |
| 15 | If they performed quantitative synthesis did the review authors carry out an adequate investigation of publication bias (small‑study bias) and discuss its likely impact on the results of the review? | Yes/Partial Yes/No |
| 16 | Did the review authors report any potential sources of conflict of interest, including any funding they received for conducting the review? | Yes/Partial Yes/No |

Notes: Quality Grade and Criteria: High: All 7 critical items are scored "Yes"; no more than 2 non-critical items are "not fully fulfilled"; Moderate: 5–6 critical items are "Yes"; 3–4 non-critical items are "not fully fulfilled"; Low: 3–4 critical items are "Yes"; ≥5 non-critical items are "not fully fulfilled"; Critically Low: ≤2 critical items are "Yes"; no limit on non-critical items..

Table S5. Data extract of included articles.

| **Title** | **Author, Year** | **LE synthesis type** | **AI/Semi-automated involvement** | **The phases of LE synthesis** | **Developing/Application** | **Precision/Recall/F1 score** | **Challenge** |
| --- | --- | --- | --- | --- | --- | --- | --- |
| Ensemble of deep learning language models to support the creation of living systematic reviews for the COVID-19 literature [31] | Knafou et al., 2023 | Living evidence synthesis | AI | Database searching and eligibility | Application tool | The ensemble attained an F1 score of 89.2 for the classification task’s class level, obtained the highest recall@3 of 89% and precision@3 of 29.69%. | The models are trained and evaluated using a dataset that comprises solely one living evidence knowledge base. |
| Vascular image registration techniques: A living review [32] | Matl et al., 2017 | Living evidence synthesis | Semi-automated | Data extraction/collection and risk of bias assessment | Application tool | Not mentioned. | Not reported the challenge about AI/semi-automated tools. |
| Data extraction methods for systematic review (semi)automation: A living systematic review [33] | Schmidt et al., 2021 | Living evidence synthesis | AI, Semi-automated | Database searching and eligibility, Data extraction/collection and risk of bias assessment | Application tool | Not mentioned. | Not reported the challenge about AI/semi-automated tools. |
| A real-world evaluation of the implementation of NLP technology in abstract screening of a systematic review [28] | Perlman-Arrow et al., 2023 | Living evidence synthesis | Semi-automated | Database searching and eligibility | Application tool | The NLP-assisted abstract screening tool achieved a precision of 92.10% and a recall of 90.00% to 100%. Additionally, it achieved an F1 score of 0.926. | The tool faces challenges in assessing its impact due to the absence of a gold standard, uniform treatment of false negatives, lack of comprehensive downstream analysis, and difficulty quantifying individual feature contributions. |
| Testing a Machine Learning Tool for Facilitating Living Systematic Reviews of Chronic Pain Treatments [34] | Chou et al., 2020 | Living evidence synthesis | AI | Database searching and eligibility | Application tool | Using AI had 96% to 100% sensitivity and had a precision rate of only 0.2%-8%. | The machine learning classifiers face challenges such as low precision, varying efficiency across topics, imbalanced data issues, and the need for more data to confirm performance in update searches for facilitating LSRs of chronic pain treatments. |
| Crowd-sourcing and automation facilitated the identification and classification of randomized controlled trials in a living review [35] | Kamso et al., 2023 | Living evidence synthesis | AI, Semi-automated | Database searching and eligibility | Application tool | Machine learning had a sensitivity of 99.3%. | The main challenges and limitations of machine learning classifiers for LSRs of chronic pain treatments include the lag in indexing new studies in MEDLINE and the trade-off between precision and recall, with text word searches having high sensitivity but low precision, and classifiers improving recall but still having low precision. |
| Quantifying absolute benefit for adjuvant treatment options in renal cell carcinoma: A living interactive systematic review and network meta-analysis [36] | Riaz et al., 2022 | Living meta-analysis | Semi-automated | Database searching and eligibility, Data extraction/collection and risk of bias assessment, Synthesis and analysis | Application tool | Not mentioned. | Not reported the challenge about AI/semi-automated tools. |
| Optimising process and methods for a living systematic review - 30 search updates and three review updates later [37] | Butler et al., 2023 | Living evidence synthesis | Semi-automated | Database searching and eligibility | Application tool | Not mentioned. | Not reported the challenge about AI/semi-automated tools. |
| A Living, Interactive Systematic Review and Network Meta-analysis of First-line Treatment of Metastatic Renal Cell Carcinoma [38] | Riaz et al., 2021 | Living meta-analysis | Semi-automated | Database searching and eligibility, Data extraction/collection and risk of bias assessment, Synthesis and analysis | Application tool | Not mentioned. | Not reported the challenge about AI/semi-automated tools. |
| Adjuvant Tyrosine Kinase Inhibitors in Renal Cell Carcinoma: A Concluded Living Systematic Review and Meta-Analysis [39] | Riaz et al., 2021 | Living meta-analysis | Semi-automated | Database searching and eligibility, Synthesis and analysis | Application tool | Not mentioned. | Not reported the challenge about AI/semi-automated tools. |
| Systematic online living evidence summaries: emerging tools to accelerate evidence synthesis [40] | Hair et al., 2023 | Living evidence synthesis | AI, Semi-automated | Database searching and eligibility, Data extraction/collection and risk of bias assessment, Synthesis and analysis | Developing tool | Not mentioned. | The automated approach faces challenges in accurately processing unstructured preclinical research data, handling missing abstracts/full-texts, and accurately detecting PICO elements, necessitating human input for quality assurance. |
| In a pilot study, automated real-time systematic review updates were feasible, accurate, and work-saving [41] | Marshall et al., 2023 | Living meta-analysis | AI | Database searching and eligibility, Data extraction/collection and risk of bias assessment, Synthesis and analysis, Publication update | Application tool | RobotReviewer LIVE had 100% recall and 55% precision. | RobotReviewer LIVE faces challenges in performance variability for complex reviews, limited article types, data source constraints, and lack of validation beyond the current evaluation case study. |
| Utilizing Artificial Intelligence to Manage COVID-19 Scientific Evidence Torrent with Risklick AI: A Critical Tool for Pharmacology and Therapy Development [42] | Haas et al., 2021 | Living evidence synthesis | AI | Database searching and eligibility, Data extraction/collection and risk of bias assessment | Developing tool | Risklick AI had an average recall of 99.25%, a precision of 96.07%, and an F1 score of 97.59%. | Not reported the challenge about AI/semi-automated tools. |
| Using a Secure, Continually Updating, Web Source Processing Pipeline to Support the Real-Time Data Synthesis and Analysis of Scientific Literature: Development and Validation Study [43] | Vaghela et al., 2021 | Living evidence synthesis | AI | Database searching and eligibility, Data extraction/collection and risk of bias assessment, Synthesis and analysis | Application tool | Not mentioned. | REDASA faces challenges in maintaining consistent quality ratings, scaling curation efforts, and ensuring corpus representativeness across study designs and methodologies. |
| A Data-driven Living Review for Pharmacogenomic Decision Support in Cancer Treatment [44] | Karakülah et al., 2012 | Living evidence synthesis | Semi-automated | Data extraction/collection and risk of bias assessment, Synthesis and analysis | Application tool | Not mentioned. | Not reported the challenge about AI/semi-automated tools. |
| Feasibility study for interactive reporting of network meta-analysis: experiences from the development of the MetaInsight COVID-19 app for stakeholder exploration, re-analysis and sensitivity analysis from living systematic reviews [45] | Xin et al., 2022 | Living meta-analysis | Semi-automated | Data extraction/collection and risk of bias assessment, Synthesis and analysis | Developing tool | Not mentioned. | MetaInsight COVID-19 confronts limitations in handling individual patient data, facilitating user interaction, integrating risk of bias and GRADE assessments, and managing type-1 error rates in living systematic reviews with network meta-analysis. |
| metaCOVID: A web-application for living meta-analyses of COVID-19 trials [46] | Evrenoglou et al., 2023 | Living meta-analysis | Semi-automated | Database searching and eligibility, Data extraction/collection and risk of bias assessment, Synthesis and analysis | Developing tool | Not mentioned. | The challenges of metaCOVID include database dependency, lack of data export, RCT-only meta-analysis, predefined subgroup/sensitivity analyses, and potential misuse due to the ease of complex analysis without cautious interpretation. |
| Versioning computer-interpretable guidelines: Semi-automatic modeling of 'Living Guidelines' using an information extraction method [47] | Kaiser et al., 2009 | Living evidence synthesis | Semi-automated | Data extraction/collection and risk of bias assessment | Developing tool | Not mentioned. | Not reported the challenge about AI/semi-automated tools. |
| Dynameta: A dynamic platform for ecological meta-analyses in R Shiny [48] | Skinner et al., 2023 | Living meta-analysis | Semi-automated | Synthesis and analysis | Developing tool | Not mentioned. | The challenges of Dynameta include promoting non-compliant meta-analyses, lack of protocol enforcement, and the need for cautious interpretation due to type II error risk, high heterogeneity, and reduced statistical power from subsetted data analysis. |
| Evidence surveillance for a living clinical guideline: Case study of the Australian stroke guidelines [49] | McDonald et al., 2023 | Living evidence synthesis | AI, Semi-automated | Database searching and eligibility, Synthesis and analysis | Developing tool | Not mentioned. | Not reported the challenge about AI/semi-automated tools. |
| Cost-effectiveness of Microsoft Academic Graph with machine learning for automated study identification in a living map of coronavirus disease 2019 (COVID-19) research [50] | Shemilt et al., 2024 | Living evidence synthesis | AI, Semi-automated | Database searching and eligibility | Application tool | MAG workflows incorporating machine learning classifiers have recalls in the range of 0.94-0.99 and precision in the range of 0.50-0.86. | Not reported the challenge about AI/semi-automated tools. |
| Living review framework for better policy design and management of hazardous waste in Australia [51] | Le-Khac et al., 2024 | Living evidence synthesis | AI | Database searching and eligibility, Data extraction/collection and risk of bias assessment | Application tool | The best model, XGBoost, had an F1 score of 0.87. | Not reported the challenge about AI/semi-automated tools. |
| Collaborative Large Language Models for Automated Data Extraction in Living Systematic Reviews [52] | Khan et al., 2024 | Living evidence synthesis | AI | Data extraction/collection and risk of bias assessment | Application tool | From concordant responses in the prompt set, the collaborative LLM had average precision of 1.00, recall of 0.96, and F1 score of 0.98; in the test set, it had average precision of 1.00, recall of 0.92, and F1 score of 0.96. | Not reported the challenge about AI/semi-automated tools. |
| A Systematic Online Living Evidence Summary of experimental Alzheimer’s disease research [53] | Hair et al., 2024 | Living evidence synthesis | AI | Database searching and eligibility, Data extraction/collection and risk of bias assessment, Synthesis and analysis | Application tool | Machine learning classifiers had an average precision rate of about 84.5%, average F1 score of about 89.6%, and average sensitivity of 95.1%. | AD-SOLES pipelines and dashboards require ongoing maintenance to ensure that they remain available, accessible and up-to-date. |

Notes: LE, Living Evidence; AI, Artificial Intelligence; MAG, Microsoft Academic Graph.

Table S6. Characteristics of excluded studies.

| **Title** | **Author, Year** | **Reason for exclusion** |
| --- | --- | --- |
| The Development of a Living Knowledge System and Implications for Future Systematic Searching [54] | Grbin et al., 2022 | Not LE synthesis |
| PMU118 USE OF COMPUTER-ASSISTED METHODS TO REALIZE THE CONCEPT OF A LIVING SYSTEMATIC REVIEW VIA AN ONLINE PLATFORM [55] | Hearnden et al., 2019 | Conference abstract with insufficient data |
| metaCOVID: An R-Shiny application for living meta-analyses of COVID-19 trials [56] | Evrenoglou et al., 2021 | Preprint (published version already included) |
| A narrative review of recent tools and innovations toward automating living systematic reviews and evidence syntheses [9] | Schmidt et al., 2023 | Not LE synthesis |
| Developing an Incivility Dictionary for German Online Discussions - a Semi-Automated Approach Combining Human and Artificial Knowledge [57] | Stoll et al., 2023 | Not LE synthesis |
| Living Systematic Reviews: An Emerging Opportunity to Narrow the Evidence-Practice Gap [5] | Elliott et al., 2014 | Not relevant to AI/semi-automated tools used in LE synthesis |
| Angiotensin-converting-enzyme inhibitors and angiotensin II receptor blockers for COVID-19: A living systematic review of randomized clinical trials [58] | Meza et al., 2021 | Not relevant to AI/semi-automated tools used in LE synthesis |
| Macrolides for the treatment of COVID-19: a living, systematic review [59] | Verdejo et al., 2020 | Not relevant to AI/semi-automated tools used in LE synthesis |
| Vitamin C for COVID-19: A living systematic review [60] | Baladia et al., 2020 | Not relevant to AI/semi-automated tools used in LE synthesis |
| Cell-based therapies for COVID-19: A living, systematic review [61] | Rada et al., 2020 | Not relevant to AI/semi-automated tools used in LE synthesis |
| LOCATE: a prospective evaluation of the value of Leveraging Ongoing Citation Acquisition Techniques for living Evidence syntheses [62] | Gates et al., 2021 | Not relevant to AI/semi-automated tools used in LE synthesis |
| Convalescent plasma or hyperimmune immunoglobulin for people with COVID-19: a living systematic review [63] | Piechotta et al., 2021 | Not relevant to AI/semi-automated tools used in LE synthesis |
| Remdesivir for the treatment of COVID-19: a living systematic review [64] | Verdugo-Paiva et al., 2020 | Not relevant to AI/semi-automated tools used in LE synthesis |
| Dynamic meta-analysis: a method of using global evidence for local decision making [65] | Shackelford et al., 2021 | Not relevant to AI/semi-automated tools used in LE synthesis |
| Lopinavir / ritonavir for COVID-19: A living systematic review [66] | Verdugo-Paiva et al., 2020 | Not relevant to AI/semi-automated tools used in LE synthesis |
| Convalescent plasma for people with COVID-19: a living systematic review [67] | Iannizzi et al., 2023 | Not relevant to AI/semi-automated tools used in LE synthesis |
| Outpatient Treatment of Confirmed COVID-19: A Living, Rapid Evidence Review for the American College of Physicians (Version 2) [68] | Sommer et al., 2023 | Not relevant to AI/semi-automated tools used in LE synthesis |
| COVID-19 Living Overview of Evidence repository is highly comprehensive and can be used as a single source for COVID-19 studies [69] | Verdugo-Paiva et al., 2022 | Not relevant to AI/semi-automated tools used in LE synthesis |
| Online Research Topic Modeling and Recommendation Utilizing Multiview Autoencoder-Based Approach [70] | Paul et al., 2024 | Not relevant to AI/semi-automated tools used in LE synthesis |
| Living systematic review: new inputs and challenges [71] | Vergara-Merino et al., 2020 | Not relevant to AI/semi-automated tools used in LE synthesis |
| Living systematic reviews in rehabilitation science can improve evidence-based healthcare [72] | Elbers et al., 2021 | Not relevant to AI/semi-automated tools used in LE synthesis |
| Diagnostics and treatments of COVID-19: two-year update to a living systematic review of economic evaluations [73] | Elvidge et al., 2023 | Not relevant to AI/semi-automated tools used in LE synthesis |
| Infographic. Comparative effectiveness of treatments for patellofemoral pain: a living systematic review with network meta-analysis [74] | Winters et al., 2021 | Not relevant to AI/semi-automated tools used in LE synthesis |

Table S7. Results of methodological quality assessment using the JBI Critical Appraisal Checklist for Textual Evidence: Narrative.

| **Author, Year** | **Questions** | | | | | | **Scores** | **Narrative appraisal** |
| --- | --- | --- | --- | --- | --- | --- | --- | --- |
|  | **Q1** | **Q2** | **Q3** | **Q4** | **Q5** | **Q6** |  |  |
| Hair et al., 2023 [40] | Y | Y | Y | Y | Y | N | 5/6 | Exclude |
| Xin et al., 2022 [45] | Y | Y | Y | Y | Y | N | 5/6 | Exclude |
| Kaiser et al., 2009 [47] | Y | Y | Y | Y | Y | N | 5/6 | Exclude |
| Skinner et al., 2023 [48] | Y | Y | Y | Y | Y | N | 5/6 | Exclude |
| McDonald et al., 2023 [49] | Y | Y | Y | Y | Y | Y | 6/6 | Include |

Notes: Y=Yes; N=No. Exclude: indicates that the article did not meet the JBI Narrative‑type classification criteria; however, they still satisfy the inclusion criteria for AI applied in LE synthesis.

Table S8. Results of methodological quality assessment using AMSTAR 2.

| **Author, Year** | **Items** | | | | | | | | | | | | | | | | **Scores** | **AMSTAR 2 rating** |
| --- | --- | --- | --- | --- | --- | --- | --- | --- | --- | --- | --- | --- | --- | --- | --- | --- | --- | --- |
|  | **Item 1** | **Item 2** | **Item 3** | **Item 4** | **Item 5** | **Item 6** | **Item 7** | **Item 8** | **Item 9** | **Item 10** | **Item 11** | **Item 12** | **Item 13** | **Item 14** | **Item 15** | **Item 16** |  |  |
| Matl et al., 2017 [32] | 1 | 1 | 1 | 0 | 0 | 0 | 0 | 1 | 0 | 1 | 1 | 1 | 1 | 1 | 1 | 1 | 11 | Low |
| Schmidt et al., 2021 [33] | 1 | 1 | 1 | 1 | 1 | 0 | 0 | 1 | 1 | 1 | 1 | 1 | 1 | 1 | 1 | 1 | 14 | Moderate |
| Riaz et al., 2022 [36] | 1 | 1 | 1 | 1 | 1 | 1 | 0 | 1 | 1 | 0 | 1 | 0 | 1 | 1 | 0 | 1 | 12 | Moderate |
| Butler et al., 2023 [37] | 1 | 1 | 1 | 1 | 1 | 1 | 0 | 1 | 1 | 1 | 1 | 1 | 1 | 1 | 1 | 1 | 15 | Moderate |
| Riaz et al., 2021 [38] | 1 | 1 | 1 | 1 | 1 | 1 | 0 | 1 | 1 | 0 | 1 | 0 | 1 | 1 | 0 | 1 | 12 | Moderate |
| Riaz et al., 2021 [39] | 1 | 1 | 1 | 1 | 1 | 1 | 0 | 1 | 1 | 0 | 1 | 0 | 1 | 1 | 0 | 1 | 12 | Moderate |
| Karakülah et al., 2012 [44] | 1 | 1 | 1 | 1 | 1 | 1 | 0 | 1 | 1 | 1 | 1 | 1 | 1 | 1 | 1 | 1 | 15 | Moderate |
| Evrenoglou et al., 2023 [46] | 1 | 1 | 1 | 1 | 1 | 1 | 0 | 1 | 1 | 1 | 1 | 1 | 1 | 1 | 1 | 1 | 15 | Moderate |
| Khan et al., 2024 [52] | 1 | 1 | 1 | 1 | 1 | 1 | 0 | 1 | 1 | 1 | 1 | 1 | 1 | 1 | 1 | 1 | 15 | Moderate |

Notes: 1=Yes; 0=Partial Yes or No. (See AMSTAR 2 rating criteria in Table S3 notes for details.)
