# Supplementary figures and images for: The Phases of Living Evidence Synthesis Using AI: Living Evidence Synthesis (Version 1)
Source: J Med Internet Res. 2026 Jan 27;28:e76130. doi: 10.2196/76130 (PMC12842881; doi:10.2196/76130)

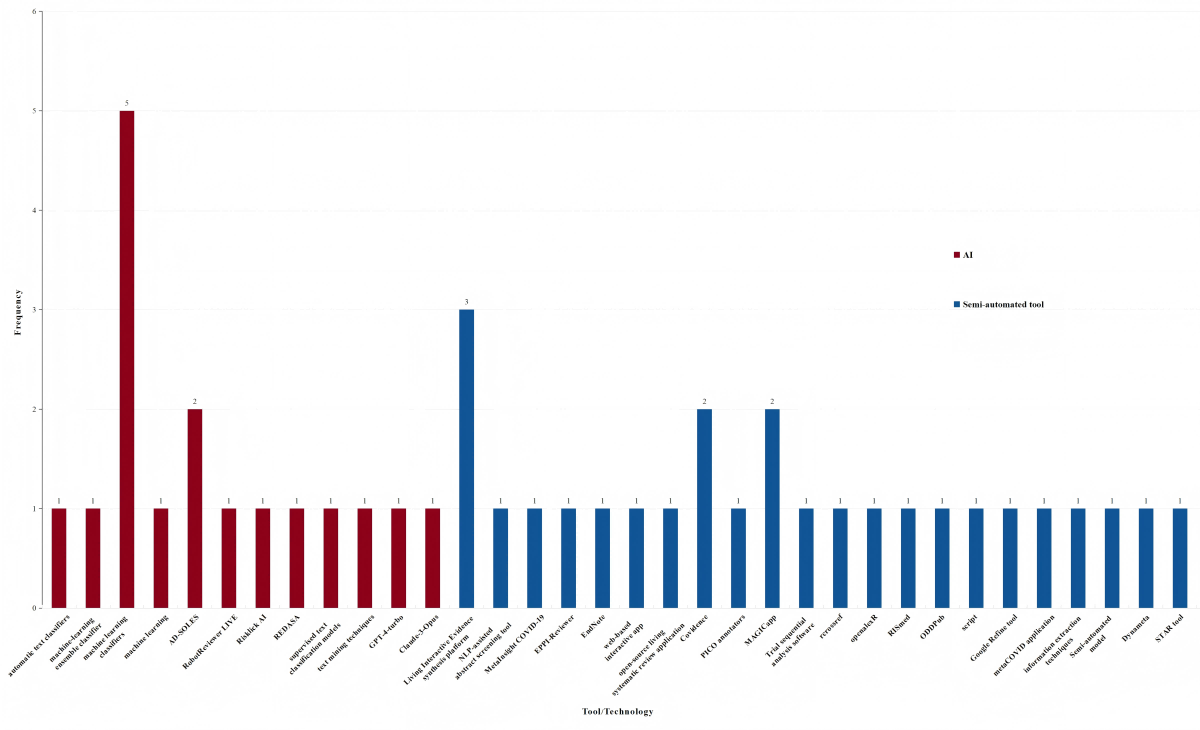

Supplement: Multimedia Appendix 2 [file jmir-v28-e76130-s002.png]
